# Multi-Organ Increase in Norepinephrine Levels after Central Leptin Administration and Diet-Induced Obesity

**DOI:** 10.3390/ijms242316909

**Published:** 2023-11-29

**Authors:** Daniela Fernandois, María Jesús Vázquez, Alexia Barroso, Alfonso H. Paredes, Manuel Tena-Sempere, Gonzalo Cruz

**Affiliations:** 1 Center for Neurobiochemical Studies in Endocrine Diseases, Laboratory of Neurobiochemistry, Department of Biochemistry and Molecular Biology, Faculty of Chemistry and Pharmaceutical Sciences, Universidad de Chile, Santiago 7820436, Chile; daniela.fernandois@inserm.fr (D.F.); aparedes@ciq.uchile.cl (A.H.P.); 2 Department of Cell Biology, Physiology and Immunology, University of Córdoba, 14004 Cordoba, Spain; bc2vavim@uco.es (M.J.V.); alexia_siver@hotmail.com (A.B.); fi1tesem@uco.es (M.T.-S.); 3 Centro de Investigación Biomédica en Red Fisiopatología de la Obesidad y Nutrición, Instituto de Salud Carlos III, 28029 Madrid, Spain; 4 Instituto Maimónides de Investigación Biomédica de Córdoba, Hospital Universitario Reina Sofia, 14004 Cordoba, Spain; 5 Instituto de Fisiología, Facultad de Ciencias, Universidad de Valparaíso, Valparaiso 2360102, Chile

**Keywords:** leptin, norepinephrine, sympathetic nervous system, ovary, liver, fat

## Abstract

Autonomic innervation is important to regulate homeostasis in every organ of the body. The sympathetic nervous system controls several organs associated with metabolism and reproduction, including adipose tissue, the liver, and the ovaries. The sympathetic nervous system is controlled within the central nervous system by neurons located in the hypothalamus, which in turn are regulated by hormones like leptin. Leptin action in the hypothalamus leads to increased sympathetic activity in the adipose tissue. In this short report, we propose that leptin action in the brain also controls the sympathetic innervation of other organs like the liver and the ovary. We performed two experiments: We performed an intracerebroventricular (ICV) injection of leptin and measured norepinephrine levels in several organs, and we used a validated model of overnutrition and obesity to evaluate whether an increase in leptin levels coexists with high levels of norepinephrine in the liver and ovaries. Norepinephrine was measured by ELISA in adipose tissue and by HPLC-EC in other tissues. Leptin was measured by ELISA. We found that the ICV injection of leptin increases norepinephrine levels in several organs, including the liver and ovaries. Also, we found that diet-induced obesity leads to an increase in leptin levels while inducing an increase in norepinephrine levels in the liver and ovaries. Finally, since hyperactivity of the sympathetic nervous system is observed both in non-alcoholic fatty liver disease and polycystic ovary syndrome, we think that an increase in norepinephrine levels induced by hyperleptinemia could be involved in the pathogenesis of both diseases.

## 1. Introduction

In recent years, a wealth of evidence has emerged to support the close interplay between metabolism and reproduction. For example, hormones associated with metabolism and the body’s energy balance, such as leptin, glucagon-like peptide-1 (GLP-1), ghrelin, and insulin, regulate the secretion of gonadotropin-releasing hormone (GnRH) and, consequently, luteinizing hormone (LH) secretion and reproduction [1,2,3]. Metabolic diseases, such as obesity, influence the levels and actions of these hormones, thereby affecting reproduction. Consequently, an understanding of the integrative mechanisms connecting metabolism and reproduction has become key for fighting two serious modern pathologies that frequently co-occur: obesity and infertility. In this regard, mounting evidence demonstrates that leptin, by controlling the central regulation of metabolism and energy balance, is essential for the onset of puberty and ovarian cyclicity, as well as sex hormone secretion in both sexes [1].

Leptin is a peripheral hormone, released mostly from white adipose tissue (WAT), whose blood levels are proportional to the amount of body fat stored [4]. The main site of action of leptin in the brain is the hypothalamus [5], where it decreases food intake and increases energy expenditure [6]. Interestingly, the centrally mediated effect of leptin on energy expenditure depends on the activation of the sympathetic nervous system (SNS) that innervates WAT and brown adipose tissue (BAT), among many organs. In WAT, the sympathetic neurotransmitter norepinephrine (NE) induces hormone-sensitive lipase, thus increasing lipolysis, while in BAT, NE is essential to activate the expression of the uncoupling protein-1 (UCP-1), thus leading to thermogenesis [7]. In the liver, NE action through beta-adrenergic receptors regulates both gluconeogenesis and lipogenesis [8].

In addition, leptin has been recognized to play a fundamental role in reproduction due to its role in puberty, controlling ovarian function [9] and modulating gonadotropin-releasing hormone (GnRH) secretion from the hypothalamus via indirect pathways [10]. Interestingly, both the onset of puberty and ovarian function have also been shown to be directly controlled by sympathetic fibers carrying NE and innervating the ovaries [11,12,13,14]. NE action in the ovaries modulates steroid hormone secretion and follicle development through its action on β-adrenergic receptors within the ovarian follicles.

Both the ovaries and the liver are innervated by postganglionic sympathetic nerves originating from the celiac ganglion [15,16]. Within the central nervous system (CNS), the hypothalamus is the main brain area controlling the activity of sympathetic nerves. Specifically, the ventromedial nucleus of the hypothalamus (VMH) plays an essential role in activating the sympathetic innervation of the liver [16], while the activation of the paraventricular nucleus of the hypothalamus (PVN) leads to increased sympathetic activity in the ovaries [15,17]. Both nuclei are activated by the action of leptin.

In this short report, we aimed to unravel whether subchronic central leptin administration increases sympathetic innervation, using NE content in peripheral tissues as an indicator. We sought to first observe the NE content in different tissues in which SNS function has been well established, including the ovaries and liver. Furthermore, since bodily fat stores of WAT are known to differ in composition, we measured sympathetic NE content differed in different WAT stores in response to a central administration of leptin. Finally, because both the ovaries and the liver are innervated by sympathetic fibers [8,16], we aimed to determine if increases in leptin due to diet-induced obesity during infancy and development might simultaneously increase NE levels in the liver and ovaries, thereby providing a tenable connection between obesity and SNS hyperactivation with two interrelated pathologies in women, such as polycystic ovary syndrome (PCOS) and non-alcoholic fatty liver disease (NAFLD) [18,19]. Our hypothesis is that both ICV leptin injection and diet-induced obesity will lead to increased levels of norepinephrine in multiple organs, including the ovaries and the liver.

## 2. Results

### 2.1. Intracerebroventricular Leptin Injection Increases the Concentration of Norepinephrine in Different Tissues

**Figure 1:**
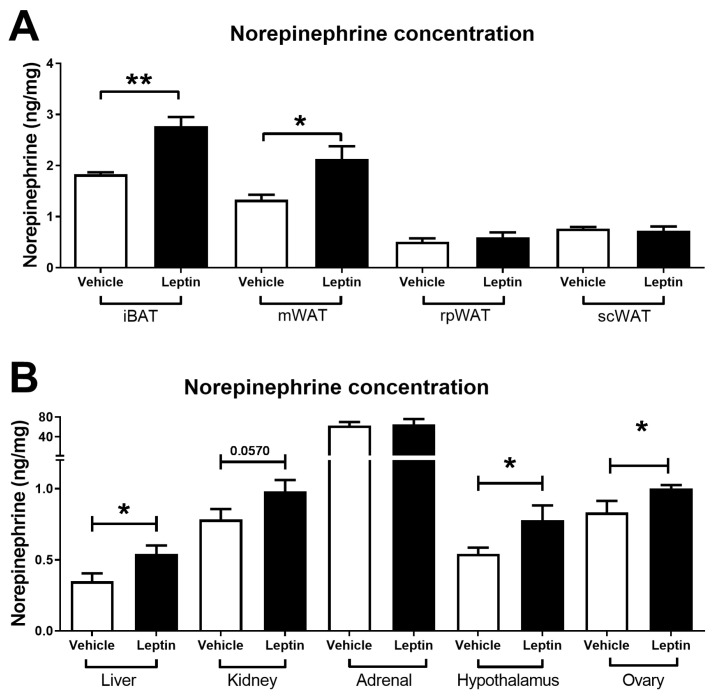
Norepinephrine concentration in different tissues after leptin injection. (**A**) Norepinephrine concentration in samples obtained from different types of adipose tissue in rats after ICV injection with leptin (Experiment 1, see Section 4). From left to right: interscapular brown adipose tissue (iBAT), mesenteric white adipose tissue (mWAT), retroperitoneal white adipose tissue (rWAT), and subcutaneous white adipose tissue (scWAT). (**B**) Norepinephrine concentration in liver, kidney, adrenal gland, hypothalamus, and ovary tissue in rats after ICV injection with leptin (Experiment 1, see Section 4). Data are shown as mean ± SEM. * = *p* < 0.05; ** = *p* < 0.01.

First, we measured NE concentrations in interscapular BAT (iBAT), subcutaneous WAT (scWAT), retroperitoneal WAT (rpWAT), and mesenteric WAT (mWAT) after 5 days of ICV leptin injection. The rationale for using a repeated dose of leptin in a short time was to simulate a sub-chronic increase in the hormone without generating leptin resistance. We observed an increase in NE concentration in iBAT and mWAT, while no changes were observed in rpWAT and scWAT (Figure 1A). Additionally, as observed in Figure 1B, ICV administration of leptin for 5 days increased the concentration of NE in the ovaries, liver, and hypothalamus. No changes were observed in the adrenal medulla; however, a clear trend of increased NE was observed in the kidney (*p* = 0.0570). In Supplementary Figure S1, we show that ICV injection of leptin for 5 days decreased food intake and body weight in female rats as a control measurement of central leptin action (Figure S1).

### 2.2. Obesity Leads to a Simultaneous Increase in Serum Leptin and Norepinephrine in the Liver and Ovaries

**Figure 2:**
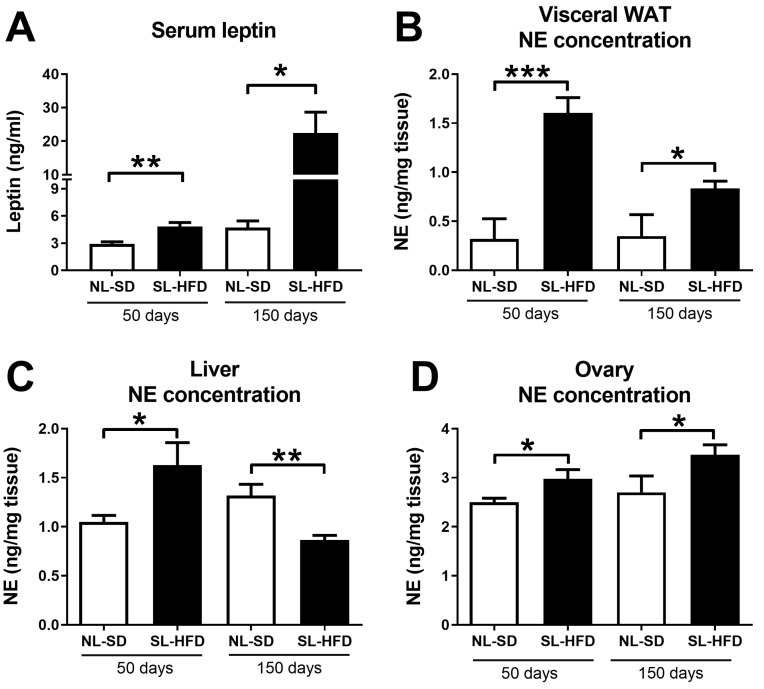
Serum leptin levels and norepinephrine concentration in different tissues in a model of diet-induced obesity. NL means “normal litter, SL means “small litter”, SD means “standard diet” and HFD means “high-fat diet”. Rats remained with their respective diets (SD or HFD) from weaning until postnatal day 50 or 150, when they were euthanized (Experiment 1, see Section 4). (**A**) Serum leptin levels in rats at 50 or 150 days old. (**B**) Norepinephrine concentration in samples obtained from mesenteric white adipose tissue (mWAT) of rats at 50 or 150 days old. Norepinephrine concentration in the liver (**C**) and in the ovaries (**D**) of rats at 50 or 150 days old. Data are shown as mean ± SEM. * = *p* < 0.05; ** = *p* < 0.01; *** = *p* < 0.001.

We employed an obesity model initiated during infancy, involving a reduction in litter size at birth, followed by the consumption of a high-fat diet (HFD) from weaning onwards (SL-HFD; small litter–high-fat diet). Control animals originated from normal litters and were provided a standard diet after weaning (NL-SD; normal litter–standard diet). Subsequently, animals were euthanized at postnatal day 50 (PND50) and postnatal day 150 (PND150). SL-HFD female rats showed higher levels of leptin at PND50 (young adulthood) and PND150 (adulthood) compared with the NL-SD (Figure 2A). Norepinephrine concentration in mWAT and the ovaries increased at PND50 and PND150 in obese rats compared to NE levels in control, lean rats (Figure 2B,C). Intriguingly, while norepinephrine concentration in the liver increased at PND50, it decreased at PND150 in obese rats compared to controls (Figure 2D).

## 3. Discussion

Weight excess and adipose tissue dysfunction are involved in the pathogenesis of several diseases. Multiple mechanisms have been proposed as a link between obesity and the development of chronic diseases, including chronic low-grade inflammation [20,21]. Obesity is involved in the pathogenesis of NAFLD [22] and also contributes to the reproductive alterations observed in PCOS [23]. Both diseases, PCOS and NAFLD, share inflammation and insulin resistance as key pathogenic mechanisms. Interestingly, increased sympathetic innervation is also involved in the development and maintenance of both PCOS and NAFLD [24,25]. Given that both ovarian and hepatic innervation are centrally regulated by the hypothalamus, we hypothesized that the central administration of leptin (ICV injection) would induce an elevation in sympathetic tone across various organs, including the ovaries and liver, as evidenced by an increase in NE content. In this brief report, we observed that obesity leads to a multi-organ increase in sympathetic NE content, particularly in the ovaries, liver, and mWAT. In this context, it is tenable that the hyperactivation of sympathetic nerves in the liver and ovaries, induced by leptin excess during obesity, could be part of the pathogenesis of NAFLD and PCOS.

Central injection of leptin for 5 days led to a simultaneous increase in NE levels in multiple tissues, including iBAT and WAT depots. Interestingly, when dissecting different WAT depots, NE levels increased only in mWAT following ICV leptin administration. This could imply a selective control of leptin in managing visceral fat depot stores. The reason for this specific impact of ICV leptin injection on iBAT and mWAT remains uncertain. However, it is established that various stimuli can differentially activate distinct white adipose tissue (WAT) depots [26]. Indeed, adipose loss in response to caloric restriction occurs preferentially from visceral depots, an effect that is prevented by the administration of the β3-AR antagonist, SR59230a [27]. The capacity of leptin to induce activation of sympathetic nerves in mWAT might be important in the pathogenesis of NAFLD since free fatty acids released from visceral fat are transported to the liver through the portal circulation.

Additionally, a central injection of leptin also led to a simultaneous increase in NE levels in the hypothalamus, liver, and ovaries. The SNS innervates the ovaries, and it is required for an adequate control of follicular development, steroidogenesis, and ovulation [11]. In fact, beta-adrenergic receptor signaling in the ovaries increases sex hormone release, similar to gonadotropins [28]. Moreover, an elevation in sympathetic tone induced by exposure to cold stress leads to a deviation of follicular growth towards abnormal cystic-like structures [29,30,31]. For instance, exposure to chronic intermittent cold stress for three to four weeks increases NE levels in the ovaries while causing a decrease in healthy antral follicles and an increase in the number of follicles with hyperthecosis (an enlarged theca layer of the follicle) [31]. These abnormal follicular structures eventually give rise to follicular cysts. Interestingly, if animals are observed for an additional four weeks without stress, follicular cysts manifest in the ovaries [29]. In another 8-week chronic stress experiment, the elevation in the number of follicles with hyperthecosis and follicular cysts is averted in animals with a lesion of the locus coeruleus—a pivotal brain nucleus regulating the sympathetic nervous system and implicated in the stress response [30]. Taking into consideration the effect of the sympathetic nervous system on ovary physiology, we propose that leptin released from adipose tissue may govern ovarian function by centrally activating the sympathetic nervous system (SNS). Similarly, an autonomic imbalance due to increased sympathetic activation is a cause of liver dysfunction [25,32]. Chronic activation of sympathetic nerves reaching the liver leads to increased gluconeogenesis and lipogenesis, thus increasing lipid accumulation and liver steatosis [33,34]. This evidence is supported by the fact that liver denervation reduces liver steatosis and improves NAFLD [33]. The absence of an effect of ICV-injected leptin on norepinephrine in the adrenal medulla means that leptin acts on sympathetic nerve pathways and does not affect the endocrine production of norepinephrine by chromaffin cells in the adrenal gland.

Since early-onset obesity produces chronic hyperleptinemia, we used SL-HFD rats to test if the elevation of endogenous leptin levels linked to obesity also increases NE content in mWAT, the ovaries, and the liver. As expected, serum leptin was increased in SL-HFD rats at PND50 (2.903 ± 0.2331 vs. 4.840 ± 0.4279 in NL-CD vs. SL-HFD, respectively) and PND150 (4.718 ± 0.7233 vs. 22.44 ± 6.229 in NL-CD vs. SL-HFD, respectively). Moreover, NE levels in mWAT, the liver, and the ovaries increased at PND50 in SL-HFD rats compared to NL-SD rats. Similar results were observed at PND150, except for liver NE levels, which decreased. Interestingly, the liver is more susceptible to metabolic challenges. Indeed, a previous report demonstrated that 20 weeks of HFD consumption leads to neuropathy and loss of sympathetic innervation in the liver [35], which is consistent with the observation of decreased NE concentration in the SL-PND150 group. PCOS has been associated with increased sympathetic activation in humans [24,36] and rodents [30,37], where sympathetic denervation restores ovulation and estrous cyclicity [38]. Therefore, sympathetic overactivation is implied in the pathogenesis of PCOS. Actually, women with PCOS have increased levels of leptin [39,40,41], a condition that is reproduced in mouse models of PCOS [42].

## 4. Materials and Methods

### 4.1. Animals

Wistar rats were bred and maintained in the dependencies of the vivarium of the University of Cordoba, Spain. All rats remained in a temperature-controlled room (22 ± 2 °C), in a 12 h light and 12 h dark cycle (light on at 08:00 a.m.), with access to food and water ad libitum. Vaginal smears were monitored each day, and only animals showing two normal estrous cycles were used. The animal protocols included in this study were approved by the Ethical Committee of the University of Córdoba; all experiments were conducted in accordance with European Union (EU) standards for the use and care of experimental animals (EU Directive 2010/63/UE, September 2010 [43]). All efforts were made to minimize the number of animals used, and all procedures were undertaken in a humane manner.

### 4.2. Experimental Design

We performed two experiments, as follows:

Experiment 1:

We placed a cannula in the lateral ventricle of the brain to allow intracerebro-ventricular (ICV) delivery in nine adult female rats. Surgery was performed under ketamine/xylazine anesthesia. The cannulae were inserted at a point that was 1 mm posterior and 1.2 mm lateral to the bregma and were lowered to a depth of 3 mm beneath the skull surface. Weight and food intake were measured daily for 21 days after surgery. After 16 days, recombinant murine leptin (7.5 µg/10 µL; ProSpec-Tany TechnoGene Ltd., Ness Ziona, Israel) or aCSF were infused into the cannula twice a day for 5 days. Then, rats were euthanized by decapitation to collect interscapular BAT (iBAT), subcutaneous adipose tissue (scWAT), retroperitoneal adipose tissue (rpWAT), and visceral mesenteric adipose tissue (mWAT), as well as the liver, kidney, and ovaries. When rats were decapitated, we dissected the hypothalamus by making a horizontal cut approximately 2 mm in depth, guided by specific anatomical landmarks described in [44,45]. These landmarks included a 1 mm cut anterior to the optic chiasm, marking the posterior border of the mammillary bodies and identifying the hypothalamic fissures. Subsequently, we fixed the rest of the brain to confirm the probe’s location through visual observation. All tissues were promptly stored at −80 °C until further processing. In this experiment, we utilized a total of 9 female rats, divided into the following groups: control animals injected ICV with aCSF (N = 4) and experimental animals injected ICV with leptin (N = 5). All rats were euthanized in the morning between 9:00 and 11:00. The rats were euthanized in the morning after the last leptin injection the previous night.

Experiment 2:

We used a previously validated model of overnutrition in which litters are reduced to 4 pups (small litters, SL) and then, from weaning onwards, they are fed with a high-fat (HF) diet (45% fat, Research Diets, New Brunswick, NJ, USA) [46,47,48,49]. Control animals were maintained as normal litters (NL, 12 pups per dam), and after weaning they were fed with a chow standard diet (SD). Rats were euthanized at postnatal day 50 (PND50, juvenile) and postnatal day 150 (PND150, adult) to evaluate plasma leptin levels and norepinephrine concentration in different tissues. Serum levels of leptin were assayed using the kit provided by EMD MILLIPORE (EZRL-83K) according to the manufactured instructions. In this experiment, we utilized a total of 28 female rats, divided into the following groups: NL-SD (N = 8) and SL-HFD (N = 8) rats at 50 days old, and NL-SD (N = 8) and SL-HFD (N = 8) rats at 150 days old. All rats were euthanized in the morning between 9 a.m. and 11 a.m.

### 4.3. Norepinephrine Concentration

For the fat adipose tissue (BAT, scWAT, rpWAT, and mWAT), NE concentration was measured using a norepinephrine ELISA kit (BA-E-5200, ImmuSmol SAS, Bordeaux, France). For the rest of the tissues, NE was quantified by high-performance liquid chromatography–coupled electrochemical detection (HPLC-EC; detector: EICOM ECD-700S). In brief, tissues were weighed in an analytical balance and homogenized manually in a glass–glass homogenizer in 200 µL of 0.2 M perchloric acid (PCA). The samples were centrifuged at 13,500× *g* at 4 °C for 15 min. The supernatant was collected and stored at −80 °C until the day of the test. Prior to the assay, an aliquot of 100 μL of the supernatant of the homogenates was mixed with 100 μL of 0.2 N PCA and filtered in 13 mm disposable filters of PVDF with a pore size of 0.22 μm (Millex™). Then, 20 μL of the filtrate was injected into the Jasco PU-2089s plus HPLC system coupled to the Jasco LC-NetII/ADC digitizer with a Kromasil 100-3.5-C18 column (AkzoNobel, Amsterdam, The Netherlands). To generate the integration of the chromatograms, the JASCO ChromPass Chromatography Data System v1.7.403.1 software was used. The mobile phase consisted of a buffer containing 0.1 M NaH_2_PO_4_, 0.14 mM octyl sulfate, 0.02% EDTA, and 1.5% acetonitrile, with a pH of 2.6. The flow rate was set to 1 mL/min. Under these conditions, the retention time was 5 min for NA. The potential of the amperometric detector was set at +750 mV.

## 5. Conclusions

Taken together, in this brief report, we have documented that in female rats, leptin action in the brain increases sympathetic nerve content of NE simultaneously in iBAT and mWAT, as well as the hypothalamus, kidney, ovaries, and liver. This is likely a reflection of an increase in sympathetic tone and activity in those tissues, leading to dysfunction. The activation of sympathetic nerves could be a key component in the pathogenesis of several diseases associated with obesity and hyperleptinemia, including NAFLD and PCOS.

### Limitations

An increase in norepinephrine concentration in the tissues could lead to different interpretations. Norepinephrine is mainly stored inside presynaptic vesicles of sympathetic fibers; thus, a high norepinephrine concentration could mean (1) an increased synthesis of norepinephrine, leading to more norepinephrine inside each vesicle, and (2) a high density of sympathetic nerves, in which the ramification of sympathetic nerves is increased. We did not measure the activity of sympathetic nerves, but both in obesity [33] and in PCOS models [37], an increase in norepinephrine tissue levels coexists with an increased activity of sympathetic nerves.

## Data Availability

The datasets collected and/or analyzed during the current study are available from the corresponding author on reasonable request.

## Supplementary Materials

The following supporting information can be downloaded at: https://www.mdpi.com/article/10.3390/ijms242316909/s1.
