# Supplementary material for: Multi-Organ Increase in Norepinephrine Levels after Central Leptin Administration and Diet-Induced Obesity
Source: Int J Mol Sci. 2023 Nov 29;24(23):16909. doi: 10.3390/ijms242316909 (PMC10706686; doi:10.3390/ijms242316909)
Supplement: Supplementary file 1 [file ijms-24-16909-s001.zip › ijms-2685613-supplementary.pdf]

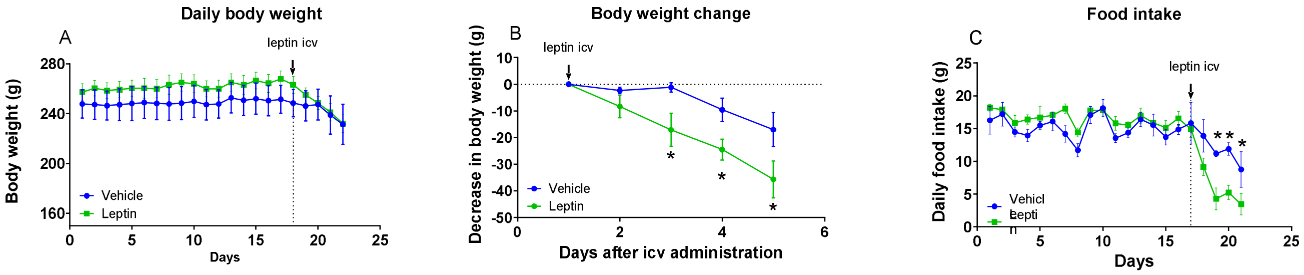

Figure S1. Leptin action for 5 days in rats. As a control, we show the action of icv leptin injection twice a day for 5 days on body weight (A), Body weight change (B) and food intake (C). Data are shown as mean ± SEM. * $p<0.05$. .
